# Mitochondrial dynamics: overview of molecular mechanisms

**DOI:** 10.1042/EBC20170104

**Published:** 2018-07-20

**Authors:** Lisa Tilokani, Shun Nagashima, Vincent Paupe, Julien Prudent

**Affiliations:** Medical Research Council Mitochondrial Biology Unit, University of Cambridge, Wellcome Trust/MRC Building, Cambridge Biomedical Campus, Hills Road, Cambridge CB2 0XY, U.K.

**Keywords:** Dynamin family, ER-Actin, Mitochondrial dynamics, Molecular Mechanisms, Regulation

## Abstract

Mitochondria are highly dynamic organelles undergoing coordinated cycles of fission and fusion, referred as ‘mitochondrial dynamics’, in order to maintain their shape, distribution and size. Their transient and rapid morphological adaptations are crucial for many cellular processes such as cell cycle, immunity, apoptosis and mitochondrial quality control. Mutations in the core machinery components and defects in mitochondrial dynamics have been associated with numerous human diseases. These dynamic transitions are mainly ensured by large GTPases belonging to the Dynamin family. Mitochondrial fission is a multi-step process allowing the division of one mitochondrion in two daughter mitochondria. It is regulated by the recruitment of the GTPase Dynamin-related protein 1 (Drp1) by adaptors at actin- and endoplasmic reticulum-mediated mitochondrial constriction sites. Drp1 oligomerization followed by mitochondrial constriction leads to the recruitment of Dynamin 2 to terminate membrane scission. Inner mitochondrial membrane constriction has been proposed to be an independent process regulated by calcium influx. Mitochondrial fusion is driven by a two-step process with the outer mitochondrial membrane fusion mediated by mitofusins 1 and 2 followed by inner membrane fusion, mediated by optic atrophy 1. In addition to the role of membrane lipid composition, several members of the machinery can undergo post-translational modifications modulating these processes. Understanding the molecular mechanisms controlling mitochondrial dynamics is crucial to decipher how mitochondrial shape meets the function and to increase the knowledge on the molecular basis of diseases associated with morphology defects. This article will describe an overview of the molecular mechanisms that govern mitochondrial fission and fusion in mammals.

## Introduction

For a long time, mitochondria have primarily been considered as the ‘powerhouse’ of the cell, producing the energy required for cell metabolism by oxidative phosphorylation (OXPHOS) [1,2]. It is now accepted that mitochondria are also involved in numerous other physiological processes such as programmed cell death, innate immunity, autophagy, redox signalling, calcium homeostasis and stem cells reprogramming [2–4]. Mitochondrial ultrastructure visualized by electron microscopy (EM) is characterized by a double membrane system. The outer mitochondrial membrane (OMM) faces the cytosol, and the inner mitochondrial membrane (IMM) protrudes into the mitochondrial matrix containing mitochondrial DNA (mtDNA). The compartment delimited by the IMM and the OMM is referred as the intermembrane space (IMS). However, the development of live cell imaging over the last 30 years has dramatically changed the concept of mitochondria being static and isolated structures. Indeed, mitochondria can modulate their morphology to create a tubular network coordinated by fission and fusion events. The balance between these two opposite processes regulates mitochondrial number, size and positioning within the cytoplasm and is referred as ‘mitochondrial dynamics’ [5].

**Figure 1:**
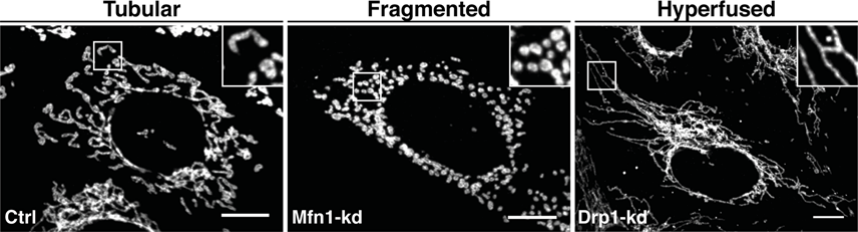
The mitochondrial morphology network Representative microscopy confocal images showing the different mitochondrial morphological aspects from control (Ctrl), Mfn1- and Drp1-Knockdown (Kd) mouse embryonic fibroblasts cells. Mitochondria are labelled with an anti-TOM20 antibody (OMM marker). Tubular, fragmented and hyperfused mitochondria are highlighted by zoomed areas (white squares); scale bars: 10 μm. Please note that the bright TOM20-positive structure in the zoom area of the Drp1-Kd is not a mitochondrial fragment but a mitochondria-derived vesicle [192].

Mitochondrial fission is characterized by the division of one mitochondrion into two daughter mitochondria, whereas mitochondrial fusion is the union of two mitochondria resulting in one mitochondrion. The deregulation of these spatio-temporal events results in either a fragmented network characterized by a large number of small round-shape mitochondria or a hyperfused network with elongated and highly connected mitochondria (Figure 1). These balanced dynamic transitions are not only required to ensure mitochondrial function but also to respond to cellular needs by adapting the network to nutrient availability and to the metabolic state of the cell [6]. Moreover, different morphological states are associated with multiple physiological and pathophysiological conditions [7]. Mitochondrial fragmentation is often linked to mitochondrial dysfunction as this morphological state predominates during elevated stress levels and cell death [8]. However, it is also observed in the phase G2/M of the cell cycle and is needed for mitochondrial motility, quality control and mtDNA inheritance [9,10]. Although still under debate, a fused mitochondrial network would allow matrix component distribution and stimulation of OXPHOS activity [11]. Mitochondrial elongation also confers protection against phagophore engulfment during autophagy triggered by nutrient starvation and is mainly associated with cell survival mechanisms [12].

**Figure 2:**
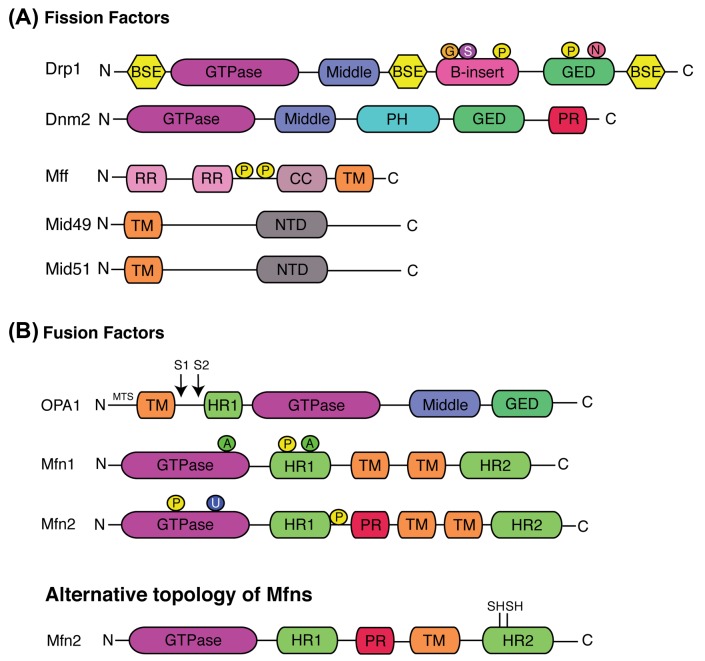
Schematic representation of the structural elements of the fission and fusion proteins, and their associated post-translational modifications Illustration of the core machinery proteins involved in (**A**) mitochondrial fission and (**B**) fusion. The classical model proposes that Mfns contain two transmembrane (TM) domains in between HR1 and HR2 domains. Alternatively, Mfns have been recently demonstrated to have only one TM that lies between the two HR domains. Cysteine residues, sensitive to oxidative stress are located in the C-terminal located in the IMS (only Mfn2 structural domains are represented but this new topology is also applicable to Mfn1). Domains are depicted in different colours. Identified location of post-translational modifications are indicated by P (Phosphorylation), N (S-nitrosylation), S (SUMOylation), G (O-GLcNAcylation), A (Acetylation) or U (Ubiquitination); BSE, bundle signalling elements; CC, coil-coil; GED, GTPase effector domain; HR, heptad repeat; MTS, mitochondrial targeting sequence; NTD, nucleotidyl transferase domains; PH, Pleckstrin homology; PR, Proline rich; RR, repeat regions; TM, transmembrane.

The main proteins composing the core machinery are large GTPase proteins belonging to the Dynamin family (Figure 2). These mechanoenzymes can oligomerize and change conformation to drive membrane remodelling, constriction, scission and/or fusion [13]. Mitochondrial constriction and scission are carried out by the Dynamin-related/-like protein 1 (Drp1) and Dynamin2 (Dnm2), respectively [14]. Mitochondrial fusion is ensured by mitofusins 1 and 2 (Mfn1 and Mfn2) and optic atrophy 1 (OPA1), which mediate OMM and IMM fusion, respectively [15]. Knockout (KO) of either of these GTPases is embryonic lethal in mice and embryonic fibroblasts derived from these mice harbour drastic mitochondrial morphology defects [16–19] (except for the Dnm2-KO mouse where mitochondrial morphology has not been investigated). The relevance of mitochondrial dynamics has also been highlighted in humans where pathogenic mutations in genes corresponding to the core fission machinery (Drp1 [20], Dnm2 [21], MFF [22] and Mid49 [23]), fusion (Mfn2 [24] and OPA1 [25,26]), and other factors involved in these events (e.g. MSTO1 [27,28], GDAP1 [29] and SLC25A46 [30,31]) have been reported.

Together, this highlights the importance of understanding how mitochondrial morphology is regulated, in order to decipher how mitochondrial shape meets the function. In this article, we will present an overview of the recent proposed mechanisms regulating mitochondrial fission and fusion in mammals.

## Molecular mechanisms of mitochondrial fusion

### Mitofusins and outer mitochondrial membrane fusion

OMM fusion is ensured by the two large GTPases homologues Mfn1 and Mfn2 in mammals, which share approximately 80% sequence similarity in humans. The Mfns orthologue, fuzzy onion (Fzo1), was originally characterized in Drosophila melanogaster [32] and is conserved from yeast [33] to human [34]. Overexpression of either Mfns leads to mitochondrial aggregation around the nucleus [35]. While Mfn1-KO induces mitochondrial fragmentation, Mfn2-KO exhibits swollen spherical mitochondria [16]. This difference can be explained by the fact that Mfn1 has been shown to have a greater guanosine triphosphate (GTP)-dependent membrane tethering activity [36]. In addition, Mfn2 is not only involved in fusion but is also a key regulator of the mitochondria-endoplasmic reticulum (ER) contact sites tethering [37,38]. Nevertheless, overexpression of Mfn1 or Mfn2 in Mfn2-KO or Mfn1-KO MEF cells, respectively, can restore mitochondrial fusion [16]. Both proteins also accumulate at contact areas between two adjacent mitochondria [35] and establish homo or heterotypic complexes leading to mitochondrial fusion [39].

**Figure 3:**
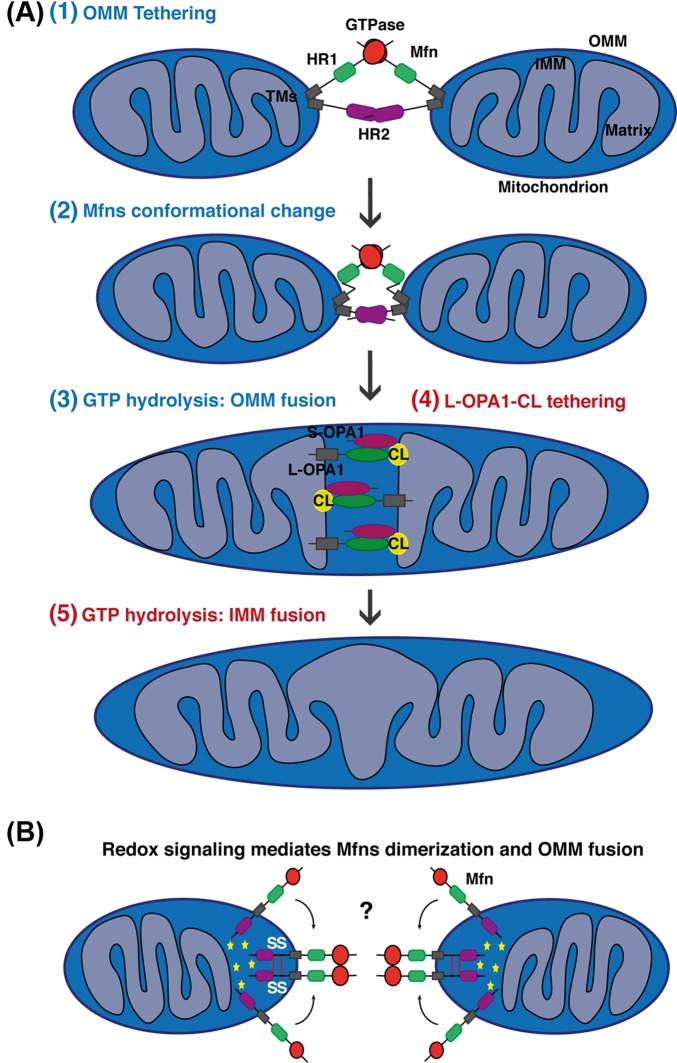
Simplified models for mitochondrial fusion in mammals (**A**) Schematic representations of mitochondrial fusion, based on the Mfns topology suggesting two TM domains with both the HR1 and HR2 domains facing the cytosol. (1) The outer membrane of two opposing mitochondria are tethered by the interaction in *trans* of the HR2 and/or GTPase domains of Mfns. GTP binding or/and hydrolysis induce Mfns conformational change leading to mitochondrial docking and to an increase of membrane contact sites. For clarity reasons, not all of the recent suggested models leading to Mfns dimerization and conformational change are highlighted in the scheme. (3) Finally, GTPase-dependent power stroke or GTP-dependent oligomerization ensure OMM fusion. The composition of the OMM in phospholipids can also regulate this process. (4) Following OMM fusion, OPA1 and CL drive IMM fusion. The interaction between OPA1 and CL on either side of the membrane tethers the two IMM, which fuse following OPA1-depedent GTP hydrolysis (5). In this model, S-OPA1 has been shown to enhance OPA1–CL interaction and fusion. Please note that after OMM and IMM fusion, Mfn2 and OPA1, as membrane-bound proteins, are still present on the different membranes but are disassembled. (**B**) Schematic representations of OMM fusion based on the new metazoan Mfns topology suggesting only one TM placing the Mfn C-terminus in the IMS. Oxidized environment in the IMS (ROS production) and increase concentration of GSSG lead to the establishment of two disulphide bonds within the IMS domain. These redox-mediated disulphide modifications induce the dimerization and oligomerization of Mfns molecules which may promote tethering or GTPase activity required for OMM fusion. Interestingly, this redox-regulated Mfns oligomerization is a dynamic and reversible process. Yellow stars indicate an oxidized environment.

Globally, mitochondrial fusion is characterized by three different steps: the tethering of two mitochondria in trans, the docking of two membranes increasing the contact surface area and decreasing the distance between the two membranes [40], and finally the fusion of the two OMM due to conformational changes induced by GTP hydrolysis [36,41] (Figure 3).

Over the last 15 years, the proposed mechanism of mitochondrial fusion by mitofusins has been based on their topology. Like yeast Fzo1 [42], it was accepted that Mfns were inserted in the OMM via two transmembrane (TM) domains separated by a short loop exposing their N-terminal region containing the GTPase and the coil-coil heptad repeat 1 (HR1) domains and their C-terminal harbouring the HR2 domain in the cytosol [34,43–45] (Figures 2 and 3A). Based on this model and some structural insights, the required mechanistic steps of fusion have been proposed (Figure 3A).

For example, it has been proposed that Mfns dimeric antiparallel *trans* interactions between apposing mitochondria are established via their HR2 domains, followed by GTP hydrolysis resulting in OMM fusion [44].

In contrast to the HR2 *trans* model, more recent structural studies conducted with a ‘minimal’ recombinant Mfn1 (internal deletion of the HR2 and generation of the predicted TM domains) revealed that the tethering is mediating through the GTPase domains [46,47]. The fusion of the adjacent membranes may then be ensured by a GTPase-dependent power stroke [47] or GTP-dependent oligomerization [46]. While crystal structures clearly reveal the GTPase binding in trans as a primary mechanism of tethering, a peptide that mimics the HR1 helix has also been shown to activate mitochondrial fusion [48]. These peptides, or smaller drugs that alter the conformation of HR1, increase mitochondrial fusion when added to cells. Based on modelling from the structures, the authors propose that these compounds interfere with HR1 binding to HR2, thereby opening the helix and promoting mitochondrial tethering and fusion [48]. These compounds were not tested in a direct mitochondrial fusion assay, so it remains possible that other mechanisms can explain their cellular effects. Finally, it has been recently proposed that the C-terminal tail of Mfn1, harbouring the HR2 domain, contains an amphipathic helix required for Mfn1 insertion and promoting mitochondrial fusion [49]. More recent work has shown that HR1, but not HR2, promotes liposome tethering and lipid mixing in reconstituted assays, hinting that this HR may destabilize the lipids to drive membrane fusion [50]. The idea that the Mfns form a larger fusion pore, visualized as a circular mitochondrial docking complex, has been suggested using cryo-electron microscopy in isolated yeast mitochondria [40]. This study has also revealed that the cycles of GTP hydrolysis are required to assemble this dynamic structure [40].

Importantly, the prediction that HR1 and HR2 both reside on the cytosolic side of the OMM was based on the established topology of the yeast orthologue Fzo1. However, this topology was recently challenged, forcing a reconsideration of the current models [51] (Figures 2 and 3B). Furthermore, phylogenetic analysis has revealed that Mfns from yeast and metazoans are highly divergent, with bioinformatics predicting a single TM domain in metazoan Mfns, with two membrane spanning regions in Mfns of the fungal clade. Classical biochemical experiments confirmed that human Mfns harbour only one TM domain, placing the ∼12 kDa C-terminal and the HR2 domain in the IMS [51]. Interestingly, this new topology has been functionally linked to the control of mitochondrial fusion by redox signalling. Indeed, two cysteines located in the HR2 domains can be oxidized by increased level of oxidized glutathione leading to the formation of disulphide bonds between two Mfns molecules and their oligomerization required for membrane fusion [51]. These results confirmed initial studies describing the role of reactive oxygen species and oxidative stress in promoting mitochondrial fusion [52–54]. This new topology is consistent with the observation that HR2 did not drive liposome tethering or fusion [50]. Given the previous assumptions that the HR2 domain resides in the cytosol, this new topology raises a number of outstanding questions, for example: Are the GTPase domain interactions in *trans* sufficient to tether two mitochondria with HR1 domain driving bilayer mixing? Do the compounds interfering with HR1 domain affect additional Mfn partners that may participate in fusion?

Together, these data highlight the requirement of a reappraisal of the current acknowledged models and further experiments based on this new model should be performed in the near future to confirm and shed light on the full mechanism of OMM fusion.

### OPA1 and inner mitochondrial membrane fusion

IMM fusion occurs downstream of OMM fusion and is mediated by the large GTPase OPA1 and specific IMM lipid components. Indeed, genetic loss of OPA1 leads to mitochondrial fragmentation whereas OPA1 overexpression induces mitochondrial elongation [55]. OPA1, originally described in the yeast model (Mgm1p) [56], is evolutionary conserved and is a complex protein with eight identified splice-variants. Its protein domain organization shares similarities with ‘classical’ dynamins (Figure 2). It is inserted within the IMM via a ∼100 residues N-terminal matrix targeting signal followed by a TM domain, exposing the majority of the protein to the IMS [57]. Despite the role of the GTPase and GTPase effector domain (GED) domains for GTP hydrolysis, the specific roles of the different domains during fusion events are not well understood. OPA1 harbours at least two sites for proteolytic cleavage, which generate shorter and soluble fragments. These cleavages are mediated by two membrane-bound metalloproteases, OMA1 [58,59] and YME1L [60,61], cleaving the protein at S1 and S2 sites, respectively. This results in at least five OPA1 fragments detectable by immunoblot where the two higher molecular weight forms are referred as L-OPA1 and the three shorter as S-OPA1. The abundance of the different OPA1 isoforms is cellular context specific and affects mitochondrial dynamics regulation. Indeed, OMA1-dependent cleavage of OPA1 is a stress response, whereas stimulation of OXPHOS induces YME1L activity. It is interesting to note that a mild mitochondrial stress leads to a stress-induced mitochondrial hyperfusion (SIMH) mechanism regulated by the Stomatin-like protein 2, Mfn1 and OPA1 and acting as a pro-survival response [62].

Initial work has described the requirement of both L- and S-OPA1 isoforms to allow mitochondrial fusion since L-OPA1 and S-OPA1 alone have only little fusion activity [61]. However, recent studies have now shown that the L-OPA1 isoform alone is sufficient to drive fusion. Indeed, L-OPA1 accumulation drives fusion during SIMH [62] and is responsible for the mitochondrial hyperfusion observed in YME1L/OMA1-DKO cells [63]. The balance between OPA1 cleavage by OMA1 and YME1L plays a crucial role in fusion regulation and the precise role of the S-OPA1 generation is not perfectly understood [64]. Indeed, initial work has shown that S-OPA1 isoform is also able to induce membrane tubulation in a liposome assay [65] and stimulation of OXPHOS induces YME1L-dependent S-OPA1 generation leading to mitochondrial fusion [66]. In contrast, mitochondrial stress-induced OPA1 cleavage by OMA1 can lead to mitochondrial fragmentation [58,59].

In mammals, OPA1 localization in only one of the two opposing mitochondria is sufficient to drive the fusion of both membranes [67]. These findings have been recently confirmed and a new model for IMM fusion regulated by OPA1 and a particular phospholipid has been proposed [68] (Figure 3A). As described later in this article, membrane lipid composition and in particular the lipid, cardiolipin (CL), play a crucial role in membrane remodelling and dynamics. CL is a mitochondria specific negatively charged lipid mainly localized in the IMM and required for the assembly and stability of large protein complexes like mitochondrial contact site and cristae organizing system (MICOS) and OXPHOS complexes [69]. Incubation of recombinant L-OPA1 with reconstituted CL-containing liposomes leads to a heterotypic interaction between L-OPA1 and CL driving membrane fusion [68]. In this model, S-OPA1 facilitates OPA1-CL binding and membranes fusion, corroborating studies performed in yeast showing the requirement of both S- and L-OPA1 isoforms as well as cardiolipin for IMM fusion [70,71]. Interestingly, these data have been validated *in cellulo* using a cell fusion assay, and suggest that the presence of OPA1 and CL on either side of the membrane can promote fusion and represents the minimal IMM fusion machinery [68]. These results also suggest that the OPA1 homotypic interaction is not involved in IMM fusion but in cristae architecture control.

Finally, OPA1-dependent IMM fusion depends on Mfn1 but not Mfn2 [62,72]. This observation raises the possible communication between the two membranes during fusion and suggests a potential interaction of Mfn1 with OPA1, a hypothesis now more plausible based on the new Mfns topology [51].

Overall, because of the complex processing of OPA1 and the lack of 3D structure of the protein, the precise mode of action of OPA1 has remained elusive. Further studies are needed to fully establish the mechanism of IMM fusion.

## Molecular mechanisms of mitochondrial fission

### Drp1 and adaptors

Mitochondrial fission is a multi-step process where the recruitment of the large GTPase Drp1 plays a crucial role. Drp1 is evolutionary conserved and its role in mitochondrial division was initially described in *Caenorhabditis elegans* [73] and yeast [74,75] before being extensively studied in mammals [76]. It is mainly a cytosolic protein, which is dynamically recruited to mitochondrial and peroxisomal membranes where it oligomerizes and drives membrane constriction in a GTP-dependent manner [14]. Indeed, genetic loss of Drp1 leads to a drastic elongation of both mitochondria and peroxisomes [77] in multiple cell lines and a variety of animal models [18,19].

Drp1 is composed of four distinct domains, an N-terminal GTPase domain followed by the middle domain, variable domain (or B-insert) and the GED in C-terminal (Figure 2). Like ‘classical dynamins’, Drp1 also contains bundle signalling elements (BSE) and stalk regions, but does not harbour the pleckstrin homology (PH) domain or the proline and arginine rich domain (PRD) at the C-terminal. The BSEs connect the GTPase domain with the stalk domain allowing Drp1 binding to membranes and subsequently its oligomerization [78].

**Figure 4:**
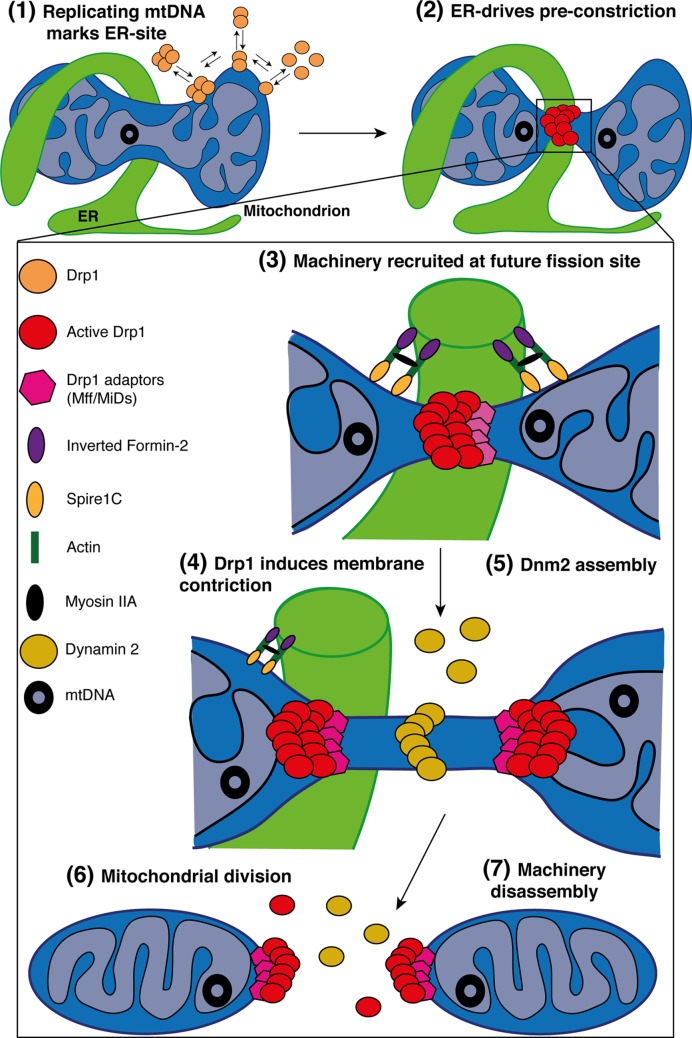
Simplified model for mitochondrial fission in mammals Schematic representation of the multi-step processes required for mitochondria division. (1) In the matrix, replication of the mtDNA marks the site for ER-recruitment. In parallel, Drp1 oligomers are in constant balance between the cytosol and mitochondria. In addition, IMM constriction occurs at mitochondria–ER contacts in a Ca^2+^-dependent process, before Drp1 oligomerization and maturation. (2) Oligomeric forms of Drp1 accumulate at ER-sites where the pre-constriction of the membrane has been initiated. (3) The zoomed area highlights the factors regulating mitochondrial division. The ER-bound INF2 and mitochondrial Spire1C induce actin nucleation and polymerization at mitochondria–ER contact sites. The Myosin IIa may ensure actin cable contraction, providing the mechanical force to drive mitochondria pre-constriction. At these sites, MFF and MiDs recruit Drp1 where it oligomerizes in a ring-like structure and (4) GTP-hydrolysis leads to conformational change, enhancing pre-existing mitochondrial constriction. The composition of the OMM in phospholipids also regulates Drp1 assembly and activity. (5) Then, Dnm2 is recruited to Drp1-mediated mitochondrial constriction neck where it assembles and terminates membrane scission, (6) leading to two daughter mitochondria. (7) The mechanisms of disassembly of the fission machinery following division remain unclear but both adaptors and Drp1 are found at both mitochondrial tips after division.

During mitochondrial division, Drp1 is recruited to the OMM where it forms a ring-like structure around mitochondria leading to the narrowing of the membrane [76,78,79] (Figure 4). Then, GTP hydrolysis enhances this membrane constriction [80] which marks a potential future site of mitochondrial scission. Assembly of Drp1 at the OMM is mediated by the central stalk (middle domain) forming Drp1-oligomeric helices starting at two different points of the membrane [78,81]. Interestingly, in contrast with the ‘common pathway’ where cytosolic Drp1 is directly recruited to a constriction site through its membrane-anchored adaptors, a ‘targeted equilibrium’ has been proposed. In this model, dimeric and oligomeric forms of Drp1 are in constant balance between the cytosol and mitochondria [82]. Mitochondria-bound Drp1 puncta can merge into a mature-sized Drp1 complex capable of moving laterally along the mitochondrial tubule, induce constriction and eventually fission [82].

As Drp1 lacks a PH domain to bind membrane phospholipids directly, its recruitment at the OMM requires adaptors proteins. In the yeast model, Dnm1 (Drp1 orthologue) is recruited to the OMM via the membrane-anchored protein fis1 [83] and two receptors Mdv1 [84] and Caf4 [85]. However, there are no obvious orthologues for Mdv1 and Caf4 in mammals and recent studies suggest that Fis1 is not involved in the fission mechanism in basal conditions [86]. Instead, the tail-anchored proteins mitochondrial fission factor (MFF) [87] and mitochondrial dynamics proteins 49 and 51 (MiD49 and MiD51) [88,89] act as receptors for Drp1 in mammals (Figures 2 and 4). On one hand, overexpression of MFF leads to a fragmented network [90] whereas MFF genetic invalidation induces mitochondrial and peroxisome elongation [87,90], accompanied by a decrease in Drp1 mitochondrial recruitment. Indeed, MFF can specifically recruit high-oligomeric forms of Drp1 *in cellulo* [91] and stimulate its GTPase [92] activity enhancing membrane constriction in liposome assay. On the other hand, MiDs overexpression leads to mitochondrial elongation due to Drp1 sequestration [88,93], whereas low levels induce mitochondrial fragmentation [94]. MiD49/51-DKO phenocopies MFF-KO, characterized by mitochondrial hyperfusion and Drp1 recruitment defects, showing a potential redundancy between MiD49 and MiD51 [86,95]. MiDs harbour a nucleotidyltransferase domain and MiD51 requires ADP as a cofactor to stimulate Drp1 oligomerization and GTPase activity [96,97]. However, *in vitro* experiments using MiD51-bound liposomes demonstrated the capacity of MiD51 to inhibit Drp1 GTPase activity [95]. While these receptors colocalize together in discrete foci with Drp1 at ER-constriction sites [86,94], MFF and MiD49/51 can act independently on Drp1 recruitment and activity [98]. Therefore, it is assumed that MFF and MiDs have distinct but complementary roles in mitochondrial fission where MiDs recruit GTP-bound state of Drp1 to facilitate oligomerization whereas MFF selectively recruits oligomeric and active forms of Drp1. These functional differences have been highlighted recently during the cell death programme [86]. Further work would shed light on the precise mechanisms of Drp1 recruitment by these adaptors.

### Final step of mitochondrial scission

Although the role Drp1 plays in membrane constriction is well described, its capacity to terminate fission has always been questioned. Recombinant Drp1 expression leads to liposome tubulation but not to their scission [99]. Moreover, cryo-EM imaging in yeast showed that the most representative diameter of Dnm1-lipid tubes constriction upon addition of GTP was 50–60 nm [100], which suggested that final scission required an additional process. Recently, the canonical protein Dnm2, initially involved in endocytic vesicle scission, has been proposed to catalyse this final step [101] (Figure 4). Like Drp1, Dnm2 is a GTPase that assembles in a collar­-like structure around the constricting lipid ‘necks’ of budding membrane-­bound vesicles [13]. Live-cell imaging experiments have shown that Dnm2 acts downstream of mitochondrial Drp1 activity, is transiently and specifically recruited to ER- and Drp1-induced constriction sites and leads to fission [101]. In addition, silencing Dnm2 induces mitochondrial elongation and the presence of highly narrow and elongated super-constriction sites. This phenotype was not rescued by re-expression of Dnm2 mutants lacking its GTPase, PH or PRD domains suggesting that activity, lipid binding and localization of Dnm2 are required for its role in mitochondrial division [101].

### Mitochondrial division occurs at ER contact sites

A groundbreaking discovery in the mitochondrial dynamics field was the discovery that the ER was required for the initial step of mitochondrial division. Indeed, high-resolution and 3D reconstructed images acquired using EM and tomography have shown that not only ER tubules make contact with mitochondria but they can also wrap around them leading to mitochondrial constriction [102] (Figure 4). This pre-constriction step is required to decrease the average mitochondrial diameter from approximately 300–500 nm to approximately 150 nm [102], to allow Drp1-oligomeric ring formation. Therefore, Drp1 and its adaptors MFF and MiD49/51 are also specifically recruited to these mitochondria–ER contact sites prior to mitochondrial division [95,102,103]. With recent evidence implicating the role of phospholipids [104] and calcium transfer [105,106] during the process, it is tempting to suggest that these ER contact sites are not just required for mitochondrial pre-constriction but also represent a signalling platform for metabolite exchange, facilitating membrane remodelling and division.

The ER-bound inverted-formin 2 (INF2) and the mitochondrial anchored formin-binding Spire1C are both actin-nucleating proteins (Figure 4). Silencing either protein leads to mitochondrial elongation and defects in actin polymerization at the mitochondria–ER interface [107,108]. At these contact sites, INF2 cooperates with Spire1C to regulate actin assembly required for mitochondrial constriction before Drp1 recruitment and oligomerization [107,108]. In addition, Myosin IIA may ensure actin cable contraction providing the mechanical force for pre-constriction site formation [109]. Furthermore, transient F-actin bursts have been observed at mitochondria just before Drp1-dependent mitochondrial division [110,111] and other proteins involved in actin cytoskeleton regulation have been shown to regulate mitochondrial fission such as cofilin [110,112], cortactin [110], Arp2/3 [110] and Septin 2 [113]. Finally, Drp1 can bind F-actin *in vitro* which stimulates its oligomerization and its GTPase activity [114]. Together, the concomitant action of the ER and actin has clearly been identified as a crucial regulator of mitochondrial division and further studies will shed light on new potential regulators and their links with other members of the fission machinery.

Since the discovery of the role of the ER in mitochondrial division and the capacity of the oligomeric form of Drp1 to move along the tubules, it was unclear how the ER identifies and marks the sites for mitochondrial division. It had already been described that mitochondrial nucleoids were localized at mitochondria–ER contact sites in yeast [115] and mammal cells [116]. Recently, using high-resolution microscopy and live cell imaging, replicating mtDNA has specifically been spatially associated at mitochondria–ER contacts and constrictions, marking future mitochondrial fission sites [117] (Figure 4), allowing mtDNA distribution to the two newly generated mitochondria. This new observation describes mtDNA replication as one of the first steps of mitochondrial division raising new questions about the regulating mechanism and how this signal coming from the matrix is transmitted to the ER.

### Constriction and division of the inner mitochondrial membrane

While the mechanisms regulating OMM constriction are well documented, the events leading to IMM constriction or division are poorly understood. Until now, no machinery has been associated with IMM division. EM analyses in *C. elegans* [73] and from rat cardiomyocytes [118] reported the potential presence of IMM constriction or division in the absence of either Drp1 or OMM constriction, which suggested that it could happen early during the mitochondrial division process. However, only very recently, an underlying mechanism for IMM constriction and possibly division has been proposed.

Coupling EM to super-resolution microscopy, two recent studies have suggested that IMM constriction is Ca^2+^-dependent and occurs at mitochondria–ER contact sites [105,106]. Stimulation of ER-induced calcium release to mitochondria leads to the constriction of the inner membrane compartment and may induce IMM division before Drp1 recruitment, therefore independently of OMM-constriction [105,106]. In addition, this phenomenon is inhibited by the loss of the mitochondrial calcium uniporter (MCU), which also leads to mitochondrial elongation. This is consistent with previous work suggesting a link between mitochondrial Ca^2+^ influx and mitochondrial fragmentation [119]. While in human Osteosarcoma cells (U2OS), IMM constriction has been attributed to the stimulation of mitochondria–ER contacts and mitochondrial calcium uptake by INF2-mediated actin polymerization [105], in neurons this mechanism is ensured by OPA1 processing [106]. Indeed, calcium entry in mitochondria induces a drop in mitochondrial membrane potential leading to the activation of OMA1 and the processing of OPA1 in S-OPA1. S-OPA1 accumulation disrupts the capacity of the MICOS complex to stabilize OMM–IMM tethering, leading to the IMM untethering and possibly constriction [106]. This proposed mode of action confirms the role previously described for S-OPA1 in fission. Indeed, S-OPA1 can localize at mitochondria–ER contact sites with the OMM fission machinery, but also overexpression enhances mitochondrial fission [120]. However, further studies need to be performed to decipher the players regulating IMM constriction and division and precisely incorporate these events in the global mitochondrial division process.

## Additional layers of mitochondrial dynamics regulation

### Membrane lipids composition in mitochondrial dynamics

Phospholipids are the major components of mitochondrial membranes and their role in membrane curvature, remodelling and regulation of mitochondrial dynamics has recently emerged. Mitochondrial membranes are mainly composed of phosphatidylcholine and phosphoethanolamine but also contain minor amounts of other phospholipids like phosphatidic acid (PA) and cardiolipin (CL), which play a major role in membrane remodelling. PA, a saturated lipid, is directly transferred from the ER to mitochondria, where it is converted into CL at the IMM [121]. A small amount of CL can be located to the OMM where CL can be converted into PA by the OMM C-anchored member of the phospholipase D family, mitoPLD [104].

Initial work has demonstrated that overexpression of mitoPLD triggered mitochondrial hyperfusion, whereas its ablation inhibited fusion and induced mitochondrial fragmentation [122]. Interestingly, hydrolysis of OMM-localized PA in diacylglycerol (DAG) or lysoPA by cytosolic mitochondrial recruited PA phosphatase (lepin 1b) [123] or phospholipase (PA-PLA1) [124], respectively, inhibits fusion induced by PA accumulation. While PA accumulation enhances Mfn1/2-dependent OMM fusion [122], CL stimulates OPA1 assembly and GTPase activity, subsequently leading to liposomes membrane tubulation [65]. As described earlier, CL plays a major role in the heterotypic interaction with OPA1, which stimulates fusion and represents the minimum machinery to drive inner membranes fusion [68].

Although Drp1 lacks a specific PH domain, it has been shown that it can interact with both CL and PA. Drp1 binding to CL via its B-insert domain drives oligomerization and stimulation of its GTPase activity enhancing constriction and tubulation of liposome membranes [92,125–129], designating CL at the OMM as a pro-fission phospholipid. Interestingly, Drp1 oligomerization and GTP hydrolysis can rearrange liposome membranes containing CL to create a constricted membrane region enriched in CL and favourable to scission [126]. In contrast, PA synthesis by the mitoPLD negatively regulates Drp1-dependent mitochondrial division [130]. *In cellulo*, Drp1 binds directly PA, via an unstructured loop in its stalk domain, at the OMM constriction sites leading to its oligomerization but to an inhibition of its GTPase activity, which results in mitochondrial hyperfusion [130,131]. Overall, these studies highlight the antagonistic roles of PA and CL microdomain formation in mitochondrial fission and fusion regulation.

### Post-translational modifications of the core components

Post-translational modifications of the core protein machinery have been extensively studied in the last 10 years (Figure 2). Drp1 phosphorylation has been the most studied and phosphorylation at serine 616 and serine 637 are considered as pro-fission and pro-fusion forms, respectively. During mitosis, Drp1 is phosphorylated by cdk1/cyclin B kinase dependent on serine 616, stimulating its oligomerization, subsequently inducing mitochondrial fission and ensuring organelle distribution to daughter cells [132]. Drp1 can also be phosphorylated by other kinases at this residue during cell death by protein kinase C (PKC) [133] and the Ca^2+^-/calmodulin-dependent kinase II (CaMKII) [134,135] and by ERK-1/2 during cancer cell invasion [136,137] and cell reprograming [138]. On the other hand, protein kinase A, recruited to mitochondria through A kinase-anchoring protein 1 (AKAP1), phosphorylates Drp1 on residue 637 inhibiting fission and protecting mitochondria from autophagosomal degradation during nutrient deprivation [139] and cell death [140,141]. Dephosphorylation of this residue is carried out by the calcium-dependent phosphatase calcineurin during cell death [140,142,143] and PGAM5 during necrosis [144]. Finally, other kinases including Rho-associated coiled coil-containing protein kinase 1 (ROCK1) [145] and glycogen synthase kinase 3β (GSK3B) [146,147] can phosphorylate Drp1 and modulate mitochondrial morphology. In addition to phosphorylation, Drp1 can be dynamically SUMOylated/deSUMOylated on multiple non-consensus sites within the B domain controlling its stable association with the membrane, fission activity and cell death [148–153]. Drp1 can also be ubiquitinated by the RING-finger ubiquitin E3 ligase MARCH5/MITOL [154] and Parkin [155]. Finally, Drp1 activity can be controlled by S-nitrolysation [156–158] (but this regulation is still controversial) and O-GluNAcylation [159] modifications in its variable domain.

In addition, Drp1 receptors can also be regulated by post-translational modifications. Indeed, MFF is a substrate of the cellular energy sensor AMP-activated protein kinase (AMPK) upon mitochondrial dysfunction and a decrease in the cytosolic ATP/AMP ratio [160]. Phosphorylation of MFF enhances Drp1 recruitment, mitochondrial fission and damaged mitochondrial degradation [160]. Finally, MiD49 is also ubiquitinated by MARCH5/MITOL leading to its proteasomal degradation [161].

Post-translational modifications of mitochondrial fusion proteins are less documented. Indeed, the IMM OPA1 protein is regulated by proteolytic cleavage as already described, and only few modifications have been associated with Mfn1/Mfn2. The activity and stability of Mfn1 is regulated by ubiquitination and acetylation. MARCH5 ubiquitinates acetylated Mfn1 promoting its proteasomal degradation during mitochondrial stress [162]. During starvation, the protein deacetylase HDAC6 binds to and deacetylates Mfn1 enhancing fusion [163]. Finally, the phosphorylation of Mfn1 in the HR1 domain by the extracellular-signal-regulated kinase (ERK) inhibits mitochondrial fusion and promotes apoptosis [164].

Mfn2 can also be ubiquitinated by the HECT-type E3 ubiquitin-ligase Huwe1 [165], the RING-between RING type E3 ubiquitin-ligase Parkin [166], and the canonical RING-finger ligase MARCH5 [167] to control its activity and stability. Indeed, PINK1-phosphorylated Mfn2 can be ubiquitinated by Parkin leading to mitophagy [166] and JNK-phosphorylated Mfn2 can be ubiquitinated by Huwe1, which leads to its degradation, facilitating fragmentation and apoptosis [165].

### Other proteins regulating mitochondrial dynamics

While most work has focused on the core GTPases that govern mitochondrial dynamics, additional factors have been identified that either directly or indirectly modify mitochondrial dynamics.

Ganglioside-induced differentiation associated protein 1 (GDAP1) and SLC25A46 are two proteins which can control mitochondrial fission. GDAP1 has been proposed to participate in fission upstream MFF and Drp1 [29]. SLC25A46 has been suggested to be the mammalian orthologue of the yeast Ugo1, a protein interacting with Fzo1 and Mgm1 to coordinate outer and inner membrane fusion processes in yeast [168,169]. However, loss of SLC25A46 in human cells leads to mitochondrial hyperfusion probably due to a deregulation of mitochondrial membrane phospholipids composition suggesting that the role of SLC25A46 seems to have evolved toward a pro-fission function [30,31]. Finally, additional evidence suggests that inner mitochondrial compartments may drive mitochondrial division. An IMM protein, mitochondrial fission process 1 (MTFP1), also called MTP18, has been involved in early step of mitochondrial division, upstream Drp1 activity [170,171]. Indeed, MTFP1 loss induces mitochondrial hyperfusion and a deregulation of Drp1 phosphorylation in an unknown mechanism [172]. This small, inner membrane protein was recently shown to be a translational target of mTOR, where protein expression was lost upon starvation or inhibition of mTOR, driving mitochondrial hyperfusion which promoted cell survival [172].

MSTO1 (Misato) is a cytoplasmic regulator of the OMM fusion machinery since it depletion leads to impaired fusion [27,28]. Finally, the reactive oxygen species modulator 1 (ROMO1) protein has been identified as a redox-regulated protein required for mitochondrial fusion and normal cristae morphology [53]. ROMO1, or Mgr2 in yeast, has a primary role in regulating the lateral release of membrane proteins transiting through the Tim23 channel during biogenesis [173]. However, under oxidative stress, ROMO1 is required for OPA1 oligomerization and ROMO1 silencing induces mitochondrial fission [53], reinforcing the interplay between redox signalling and the control of mitochondrial fusion. It is unclear whether this function in OPA1 dynamics directly relates to defects in biogenesis, or if it may have a secondary stress-related function in metazoans.

## Mitochondrial dynamics: clinical syndromes

Pathogenic mutations in genes encoding the core fission and fusion machinery components have been linked to different severe human disorders, highlighting the physiological role of mitochondrial dynamics in cell homoeostasis (Table 1). These defects are mainly associated with neuromuscular and central nervous system (CNS) clinical syndromes and they are responsible for severe disabilities and progressive clinical course.

**Table 1:** Clinical syndromes due to mutations in genes encoding fission and fusion machinery components

Gene	OMIM	Inheritance	Disease	Symptoms	Refs
*MFN2*	608507	AD	Charcot–Marie–Tooth disease type 2A	Distal limb muscle weakness and atrophy, axonal degeneration/regeneration, areflexia, distal sensory loss (pain and temperature more frequent) with or without: (a) CNS involvement (cognitive decline, spasticity, encephalopathy), (b) optic atrophy, (c) hearing loss and (d) vocal cord paresis	[24]
		AR	Charcot–Marie–Tooth disease type 2A		[174]
		AD	Hereditary motor and sensory neuropathy VIA		[178]
*OPA1*	605290	AD	Optic atrophy 1	Progressive loss of visual acuity, temporal optic nerve pallor, central scotoma with or without: (a) CNS (ataxia, spasticity, hearing loss) and (b) PNS (axonal sensorineural polyneuropathy) symptoms.	[26]
		AD	Optic atrophy plus syndrome		[25]
		AR	Behr syndrome	Early-onset optic atrophy accompanied by neurologic features, including ataxia, pyramidal signs, spasticity and mental retardation	[185]
*MSTO1*	617619	AR/AD	Myopathy and ataxia	Hand and feet muscle weakness, growth impairment, fine tremor, cerebellar hypotrophy with or without: (a) white matter hyperintensities, (b) frontal lobe atrophy and (c) mental retardation	[27] [28]
*DNM1L*	603850	AR/AD	Encephalopathy	Abnormal brain development, seizures, hepatic dysfunction, encephalopathy, dysmorphism.	[20] [187]
		AD	Optic atrophy 5	Progressive loss of visual acuity, optic nerve atrophy and central scotoma	[188]
*MFF*	614785	AR	Encephalopathy	Seizures, dysphagia, optic and peripheral neuropathy, developmental delay, microcephaly, cerebellar atrophy and basal ganglia lesions	[22]
*MIEF2*	615498	AR	Mitochondrial myopathy	Progressive muscle weakness, intermittent muscle pain and exercise intolerance	[23]
*DNM2*	602378	AD	Centronuclear myopathy 1	Slowly progressive muscle weakness.	[21]
		AD	Charcot–Marie–Tooth disease, axonal type 2M	Distal limb muscle weakness and atrophy and sensory impairment, areflexia +/-neutropenia.	[180]
		AD	Charcot–Marie–Tooth disease, dominant intermediate B		
		AR	Lethal congenital contracture syndrome 5	Polyhydramnios, decreased foetal movements, intracranial bleeding, retinal haemorrhage, joint contractures and respiratory insufficiency	
*SLC25A46*	610826	AR	Pontocerebellar hypoplasia type 1	Early onset of optic atrophy, peripheral axonal sensorimotor neuropathy, ataxia, myoclonus, cerebellar atrophy, hypotonia with variable degree of severity, age at onset and association of symptoms	[189]
		AR	Hereditary sensory motor neuropathy		[31]
		AR	Optic atrophy spectrum disorders		[30]
*GDAP1*	606598	AR	Charcot–Marie–Tooth disease type 4A	Distal limb muscle weakness and atrophy and sensory impairment, areflexia with or without: (a) axonal regeneration and (b) vocal cord paresis	[176] [175]
		AR/AD	Charcot–Marie–Tooth disease type 2K		
		AR	Charcot–Marie–Tooth disease type A		
		AR	Charcot–Marie–Tooth disease with vocal cord paresis		
*INF2*	610982	AD	Charcot–Marie–Tooth disease type E	Distal limb muscle weakness and atrophy and sensory impairment, areflexia, sensorineural hearing loss and foot drop	[177]
		AD	Focal segmental glomerulosclerosis	Proteinuria and renal failure	[179]

A non-exhaustive list of the diseases related to the principal identified mutations in genes encoding the core components of mitochondrial dynamics with associated symptoms. Abbreviations: AD, autosomal dominant; AR, autosomal recessive; CNS, central nervous system; OMIM, Online Mendelian Inheritance in Man®; PNS, peripheral nervous system.

Clinical genetics studies have identified pathogenic mutations in *MFN2* [24,174], *GDAP1* [175,176], *INF2* [177] and *DNM2* [180] as a cause of different types of Charcot–Marie–Tooth (CMT) disease, a clinically diverse group of inherited peripheral neuropathies. CMT diseases are characterized by degeneration of peripheral sensory and motor axons, causing distal sensory loss, muscle atrophy and weakness. In addition, *MFN2* has also been associated with hereditary motor and sensory neuropathy VI, with optic atrophy and vocal cord paresis as potential additional symptoms [178]. Defects in the ER-associated fission protein INF2 can also cause renal focal segmental glomerulosclerosis [177,179] while *DNM2* mutations have also been responsible of centronuclear myopathy [21] or lethal congenital contracture syndrome 5 [180].

Dominant mutations have been reported in *OPA1*, and associated with optic atrophy, the most common hereditary optic neuropathy [181]. This neuropathy is characterized by a loss of retinal ganglion cells in the optic nerve, leading to a gradual and progressive loss of vision [25,26]. It has been challenging to pinpoint the cause of the selective degradation of the optic nerve, because similarly to Mfn2, OPA1 also plays multiple roles, such as in cristae architecture and in apoptosis [182]. A subset of dominant mutations in the GTPase domain of *OPA1*, which is directly involved in IMM fusion, has been associated with dominant optic atrophy plus syndrome, defined by the development of additional symptoms such as deafness, ataxia and myopathy throughout adulthood [183,184]. Instead, recessive mutations in *OPA1* cause Behr syndrome, a complex neurological disorder characterized by early-onset optic atrophy, ataxia, spasticity and mental retardation [185]. These three distinct clinical syndromes due to *OPA1* mutations are not clearly explained by the underlying pathophysiology [186].

Mutations in pro-fusion gene *MSTO1* cause mitochondrial myopathy and ataxia [27,28] while mutations in *DRP1* lead to a severe neurological syndrome with microcephaly, abnormal brain development, optic atrophy and persistent lactic acidemia [20,187,188]. Furthermore, defects involving Drp1 adaptors, *MFF* [22] and *MIEF2* [23], have been linked to human diseases. Indeed, patients harbouring mutations in *MFF* exhibit seizures, optic neuropathy and microcephaly [22]. Patients carrying mutations in *MIEF2* suffer from myopathy, with complex I and complex IV activity deficiency in muscle [23]. Pathogenic mutations have also been associated with other regulators of mitochondrial fission. Indeed, patients carrying mutations in *SLC25A46* have been reported to present clinically heterogeneous disorders, ranging from pontocerebellar hypoplasia [189], hereditary sensory motor neuropathy [31] to optic atrophy spectrum disorder [30].

Studies on *in vivo* and *in vitro* models with defective fission/fusion machinery components have been fundamental for shedding light on the crucial role of mitochondrial dynamics in the control of cell fate decisions. However, the clinical and genetic complexity of these disorders have not been explained yet and additional studies are required to improve our understanding on the molecular basis of diseases associated with mitochondrial dynamic defects.

## Conclusions

Mitochondrial fusion and fission are crucial events and it is evident that these dynamic morphological transitions control cell fate decisions. Elucidating how these events are regulated, from a molecular but also biological point of view, represents a crucial step to the understanding of numerous human diseases. The discovery of new players which regulate these events is in constant evolution, from unexpected organelles [190] to key biological events [191], and that will continue in the following years with the development of novel microscopy technology and genetic tools.

## Summary

- Mitochondria are highly dynamic organelles that remodel their network in order to maintain their shape, distribution and size.
- The balance between fission and fusion events modulates mitochondrial morphology depending on the metabolic needs of the cell.
- The components of the core machinery regulating mitochondrial dynamics belong to the Dynamin family and these mechano-GTPases enzymes ensure these dynamic transitions.
- Mitochondrial dynamics are controlled by additional layers of regulation including the ER–actin axis, membranes lipid composition and post-translational modifications of the key proteins.
- Mitochondrial fission and fusion regulate numerous physiological functions and numerous diseases have reported abnormal mitochondrial morphology.

## Abbreviations

ADP: adenosine diphosphate
AMPK: AMP-dependent protein kinase
BDLP: bacterial dynamin-like protein
CaMKII: Ca^2+^-/calmodulin-dependent kinase II
CL: cardiolipin
Dnm2: Dynamin 2
Drp1: Dynamin-related protein 1
ER: endoplasmic reticulum
GDAP1: ganglioside-induced differentiation associated protein 1
GTP: guanosine triphosphate
HR: heptad repeats
IMM: inner mitochondrial membrane
INF-2: inverted formin-2
MFF: mitochondrial fission factor
Mfn1: mitofusin 1
Mfn2: mitofusin 2
MiD49: mitochondrial dynamic protein 49
MiD51: mitochondrial dynamic protein 51
MSTO1: mitochondrial distribution and morphology regulator 1
mtDNA: mitochondrial DNA
MTFP1: mitochondrial fission process protein 1
OMA1: overlapping with the M-AAA protease
OMM: outer mitochondrial membrane
OPA1: optic atrophy protein 1
PA: phosphatidic acid
PH: pleckstrin homology
PKC: protein kinase C
PR: proline rich
ROMO: reactive oxygen species modulator
TM: transmembrane
